# DRB1*11 allele expression and HER2 pre-existing immunity may predict benefit in breast cancer patients vaccinated with the HER2 modified AE37 peptide vaccine

**DOI:** 10.1186/2051-1426-3-S2-P427

**Published:** 2015-11-04

**Authors:** Eleftheria A Anastasopoulou, Panagiotis Tzonis, Sotirios P Fortis, Louisa Mahaira, Christoforos Vaxevanis, Alexandros Ardavanis, Elizabeth A Mittendorf, George E Peoples, Constantin N Baxevanis, Michael Papamichail, Sonia A Perez

**Affiliations:** 1 Cancer Immunology and Immunotherapy Center, Saint Savas Cancer Hospital, Athens, Greece; 2 St Savas Cancer Hospital, 1st Medical Oncology Clinic, Athens, Greece; 3 The University of Texas MD Anderson Cancer Center, Houston, TX, USA; 4 Cancer Vaccine Development Program, San Antonio, TX, USA

## Background

Identification of immune biomarkers indicating potential clinical benefit from immunotherapies may contribute to the selection of the right patient for the right therapy. We have recently reported that HLA-DRB1*11 and HLA-A*24 alleles might serve as predictive factors for immunological and clinical responses to vaccination with AE37, the Ii-Key hybrid peptide of HER2_776-790_ (AE36) in prostate cancer patients. The purpose of this study was to investigate the predictive significance of DRB1*11 allele expression in relation to immunological and clinical response in breast cancer patients vaccinated with AE37 in a randomized Phase II clinical trial.

## Methods

This trial (ClinicalTrials.gov Identifier: NCT00524277) enrolled node-positive or high risk node-negative patients with any degree of HER2 expression (IHC 1-3+ or FISH > 1.2), rendered disease-free following standard of care therapy. Patients were randomized to receive either AE37+GM-CSF (vaccine group) or GM-CSF alone (control group) in 6 monthly intradermal primary inoculations followed by 4 boosters administered every 6 months. The current analysis includes data from 55 patients enrolled and vaccinated with AE37 in Greece, where the frequency of HLA-DRB1*11 is high. HLA-typing and measurement of TGFβ levels in serum were performed using Luminex® technology. Immunologic responses were assessed *in vivo* using the delayed-type hypersensitivity (DTH) test and *in vitro* with IFN-γ ELISPOT assay.

## Results

Of the 55 vaccinated pts, 31 were found to be DRB1*11+ (56%). At baseline prior to vaccination, 22% (12 out of 55) of the vaccinated patients demonstrated pre-existing immunity against AE36 by IFN-γ release (defined as above the 75th percentile of all enrolled patients). Among patients with pre-existing AE36 immunity, the majority, 67% (8 out of 12), were found to be DRB1*11^+^. Vaccine-induced DTH and IFNγ responses, were augmented in the vast majority of DRB1*11+ patients. No correlation was observed between the pre-existing levels of serum TGFβ and the expression of the DRB1*11 allele. With a median follow up of 71 months, Kaplan-Meir analyses demonstrated a 22% and 72% relative reduction in recurrence rate (RRR) in DRB1*11^+^ patients and those with pre-existent immunity, respectively. Overall survival analysis of DRB1*11^+^ patients showed a 50% relative reduction in death rate (RRD), and a 74% RRD in patients with pre-existent immunity.

## Conclusions

Our data demonstrate both immunologic and clinical advantage of vaccination therapy with AE37 among patients expressing DRB1*11 and/or having pre-existing HER2 immunity, highlighting their potential roles as predictive biomarkers to select patients most likely to benefit from vaccination with AE37.

## Trial Registration

ClinicalTrials.gov identifier NCT00524277.

